# Growth Performance, Bone Development and Phosphorus Metabolism in Chicks Fed Diets Supplemented with Phytase Are Associated with Alterations in Gut Microbiota

**DOI:** 10.3390/ani12070940

**Published:** 2022-04-06

**Authors:** Lan Li, Xiaoyi Zhang, Jiatu Zhang, Meiling Liu, Lihong Zhao, Cheng Ji, Jianyun Zhang, Shimeng Huang, Qiugang Ma

**Affiliations:** State Key Laboratory of Animal Nutrition, College of Animal Science and Technology, China Agricultural University, Beijing 100193, China; lanli0928@163.com (L.L.); zxyxjljy@163.com (X.Z.); twinkletu2958@hotmail.com (J.Z.); 17861509728@163.com (M.L.); zhaolihongcau@cau.edu.cn (L.Z.); jicheng@cau.edu.cn (C.J.); jyzhang@cau.edu.cn (J.Z.); shimengh@cau.edu.cn (S.H.)

**Keywords:** phosphorus, phytase, growth performance, egg-laying chicks, gut microbiota

## Abstract

**Simple Summary:**

Phosphorus is a crucial component of nucleic acids, phospholipids, several coenzymes and bone, and plays numerous roles in nutrient metabolism in animals. We investigated the growth performance, bone development, phosphorus metabolism and gut microbiota changes elicited by different phosphorus levels with/without phytase in chicks during the brooding period. Low-phosphorus diets inhibited growth performance and bone development, decreased utilization of phosphorus and altered gut microbial structure and function in the brooding stage of chicks. Inclusion of phytase improved growth performance and bone development and decreased phosphorus emission. The potential mechanisms may be associated with gut microbiota reprogramming.

**Abstract:**

Phosphorus pollution caused by animal husbandry is becoming increasingly problematic, especially where decreasing and non-renewable phosphorus resources are concerned. We investigated the growth performance, bone development, phosphorus metabolism and gut microbiota changes elicited by different phosphorus levels with/without phytase in chicks during the brooding period (1–42 d). Five-hundred-and-forty (540) egg-laying chickens were assigned to six groups (0.13% NPP, 0.29% NPP, 0.45% NPP, 0.13% NPP + P, 0.29% NPP + P and 0.45% NPP + P) according to a factorial design with three non-phytate phosphorus (NPP) levels (0.13, 0.29 and 0.45%) and two phytase (P) dosages (0 and 200 FTU/kg). Chicks fed with the diet with 0.13% NPP had the lowest body weight, average daily gain, shank length, average daily feed intake and highest ratio of feed to gain, while phytase supplementation was able to mitigate the adverse effects of low-phosphorus diets on growth performance. Moreover, phosphorus metabolism was affected by different dietary NPP and phytase levels. Thus, 0.13% NPP significantly reduced serum phosphorus, while phytase supplementation significantly increased serum phosphorus. Notably, phosphorus utilization in the 0.13% NPP group was significantly decreased and the phosphorus excretion ratio was increased. Phytase supplementation significantly improved phosphorus utilization by 43.79% and decreased phosphorus emission in the 0.13% NPP group but not in the 0.29% NPP or the 0.45% NPP group. Remarkably, the alpha diversity of gut microbiota was significantly decreased in the low-phosphorus group, while phytase supplementation increased alpha diversity and improved gut microbial community and function. The LEfSe analysis revealed that several differential genera (e.g., *Bacteroides*, *norank_f__Clostridiales_vadinBB60_group* and *Eggerthella*) were enriched in the different dietary NPP and phytase levels. Furthermore, correlations between differential genera and several crucial phenotypes suggested that the enrichment of beneficial bacteria with different levels of phosphorus and phytase promoted phosphorus utilization in the foregut and hindgut. In summary, low-phosphorus diets inhibited growth performance and bone development, decreased utilization of phosphorus and altered gut microbial structure and function in the brooding stage of chicks. Finally, phytase supplementation improves growth performance and bone development and decreases phosphorus emission, and the potential mechanisms may be associated with the reprogramming of gut microbiota.

## 1. Introduction

Phosphorus is a crucial component of nucleic acids, phospholipids, several coenzymes and bone and plays numerous roles in nutrient metabolism in animals [1,2]. It is therefore consequential that the world’s phosphate rock supplies may be depleted over the next 50–100 years [3]. In general, mineral feed phosphates are frequently added to chicken diets since the quantity of accessible phosphorus supplied by plant-based feed components is deemed inadequate [4]. However, feed phosphates are produced from mined rock phosphate; therefore, it is necessary to decrease using feed phosphates to make poultry production more sustainable [5]. In addition, excessive P supplementation results in higher feed prices and higher phosphate contents in excreta [3]. This can lead to phosphorus pollution issues, such as the eutrophication of sensitive ecosystems [6,7].

The phosphorus availability of planted feed ingredients is regarded as insufficient. In a balance trial in pigs, the phosphorus availability of maize was found to be 18%, wheat 62% and triticale 52% [8]. In poultry production, phosphorus availability reported from laying hen studies varied from 23 to 53% [4]. In vegetable feedstuffs, phytic acid is an anti-nutritional constituent to block P digestion [9]. In plant seeds, phytic acid is naturally present as phytate, the principal storage form of phosphorus for mature poultry [9]. Many studies have demonstrated the usefulness of phytases in enhancing phosphorus bioavailability, thus inorganic phosphorus supplementation could be reduced and consequent phosphorus excretion minimized in animal production [10,11]. During the past decade, the inclusion of microbial phytase in poultry diets has increased remarkably, mainly due to its benefits to poultry production [12] and in response to concerns about phosphorus pollution [13].

The gut microbiome is extremely important in animal health due to its role in shaping the biochemical profile of diets [14]. Recent research reveals that calcium phosphate has a considerable impact on gut microbiota alterations caused by inulin and galacto-oligosaccharides ingestion [15]. Moreover, phytase affects the gut microbiota of chicken, which is connected to the contents of dietary phosphorus and Ca [16]. Despite increasing research into the effects of phytase on phosphorus metabolism and growth performance, the relationship between growth performance and phosphorus emission and gut microflora in chicks is rarely considered. Therefore, we hypothesized that different levels of phosphorous with phytase affect growth performance, reshape gut microbiota and reduce phosphorus pollution in layers. Hence, the goal of this trial was to explore the effects of varying doses of phosphorus and phytase on the growth performance, phosphorus metabolism and emission of phosphorus and the microflora community in chicks.

## 2. Materials and Methods

The animal experimental and sample collection were approved by the Animal Care and Use Committee of China Agricultural University (AW13301202-1-14). All procedures were conducted following the guidelines for Experimental Animals (Beijing, China).

### 2.1. Animals, Housing and Experimental Design

Five-hundred-and-forty one-day-old white egg-laying strain chicks (Jing Pink, a cultivated brand with pink eggshell and white feathers and medium body size, supplied by Yukou Poultry Co., Ltd., Beijing, China) with an average body weight (BW) of 37.55 ± 0.30 g were randomly assigned to six treatments (6 replicates of 15 birds) with three dietary NPP supplementation levels (0.45%, 0.29% and 0.13%) with/without phytase (0 FTU/kg and 200 FTU/kg). All six experimental treatment diets were prepared to meet the Chinese Feeding Standard of Chickens except for phosphorus and phytase (Table 1). Chicks were provided with feed and water ad libitum. A 23 h light schedule was applied for the initial three-day period, followed by 22 h of lighting with 2 h of darkness during the fourth and seventh days, and then an increase of 2 h of darkness each week until the chicks had reached 6 weeks of age. The temperature of the animal housing was kept at roughly 35 °C for the first week, then progressively dropped to 25 °C for the remainder of the experiment. Protocols for disinfection and immunization were followed in line with the commercial layer’s management manual. The experiment lasted 6 weeks.

### 2.2. Growth Performance and Bone Development Analysis

On days 14 and 42, the BW and shank length were measured. BW, average daily feed intake (ADFI), average daily gain (ADG), feed to gain ratio (F:G) and increment of shank length were calculated.

### 2.3. Blood Biochemical Parameters Analysis

Blood samples were obtained from the wing vein of one bird from each replicate at random in order to prepare serum samples on days 14 and 42 [17]. The concentrations of serum Ca, serum P, Albumin (ALB), and Alkaline Phosphatase (ALP) were determined by an automatic biochemical analyzer (Hitachi Co., Ltd., Tokyo, Japan) with commercial kits (Nanjing Jiancheng, China). Moreover, the contents of Bone Alkaline Phosphatase (BALP), Calcium Binding Protein (CaBP), Calcitonin (CT), Parathyroid Hormone (PTH), 1,25(OH)2D3 and 25(OH)D3 were detected by commercial ELISA kits (Nanjing Jiancheng, China).

### 2.4. Phosphorus Digestibility and Emission Analysis

Fecal digestibility was determined by the indicator method, which uses acid-insoluble ash as the endogenous indicator [18]. On days 33–35 of the experiment, the excreta were collected every 24 h and feathers and other debris were removed. Then, 5 g fresh manure was mixed with 10 mL of 10% sulfuric acid. The 3-day excrement was evenly mixed and put it into an oven at 65 °C to dry to constant weight, put indoors for moisture regain for 24 h, mashed through a 40-mesh sieve, then stored at −20 °C for further analysis.

Hydrochloric acid-insoluble ash contents were determined using the method described in a previous report [19]. Total phosphorus and Ca contents were analyzed using ICP-OES (ICP 720ES, Agilent, Santa Clara, CA, USA).

The dry matter (DM) digestibility, apparent total tract digestibility (ATTD) of phosphorus, phosphorus emission rate, phosphorus emission per kilogram of feed, daily phosphorus emission, ATTD of Ca, Ca emission rate, Ca emission per kilogram of feed and daily Ca excretion were calculated. The calculation formulas were as follows:

DM digestibility (%) = [1 − (Im × Pc)/(Id × Pd)] × 100

ATTD of P (%) = (1 − [(Im × Pc)/(Id × Pd)) × 100, where Im is the content of the acid-insoluble ash in the diet, Pc is the P content in feces, Pd is the P content in the diet and Id is acid-insoluble ash in feces.

Phosphorus excretion rate (%) = 100 − ATTD of P.

Phosphorus emission/kg of feed = total phosphorus in diet × phosphorus excretion rate.

Daily phosphorus excretion (g/d) = average daily feed intake × total phosphorus content × phosphorus excretion rate.

ATTD of Ca (%) = [1 − (Im × Cac)/(Id × Cad)] × 100, where Im is the content of the acid-insoluble ash in the diet, Cac is the Ca content in feces, Cad is the Ca content in the diet and Id is the acid-insoluble ash in feces.

Ca excretion rate (%) = 100 − ATTD of Ca.

Ca excretion emission/kg of feed = total Ca in diet × Ca excretion rate.

Daily Ca excretion (g/d) = average daily intake × total Ca content × Ca excretion rate.

### 2.5. Gut Microbial Analysis

The fresh cecal contents were collected for gut microbiota analysis. The E.Z.N.A. Stool DNA Kit was used to extract total DNA from the cecal contents of chicks, and a NanoDrop spectrophotometer was used to determine the amount and quality of the DNA. A commercial business (Majorbio, Shanghai, China) performed the Illumina MiSeq sequencing and data analytics. The V3 and V4 regions were PCR-amplified using the 338F (5′-ACTCCTACGGGAGGCAGC-3′) and 806R (5′-GGACTACHVGGGTWTCTAAT-3′) primers reported in a previous work [20]. The Illumina MiSeq paired-end sequenced (2300) platform was utilized to sequence purified amplicons, and the sequencing results were employed for bioinformatics analysis.

The methods used for intestinal microbiome analysis were consistent with a previous paper [21]. Alpha-diversity measure was analyzed using MOTHUR software (1.30.2). QIIME (1.9.1) was used to perform PCoA. The LEfSe analysis was utilized to identify the distinct bacterial populations at different phosphorus and phytase levels. Spearman correlation analysis was utilized in the Pheatmap package in R (3.3.1) to investigate the relationship between differential microbiota and growth performance, serum parameters and the P emission-related index.

### 2.6. Statistical Analysis

Except for microbiota analysis, the general linear model and two-way analysis of variance procedure in SAS 9.0 were used for analysis. After the ANOVA procedure, Tukey’s multiple comparisons test was applied for specific differences. All results were listed as means ± standard error of the mean (SEM). When the *p*-value < 0.05, differences were considered statistically significant.

## 3. Results

### 3.1. Growth Performance and Bone Development

**Figure 1:**
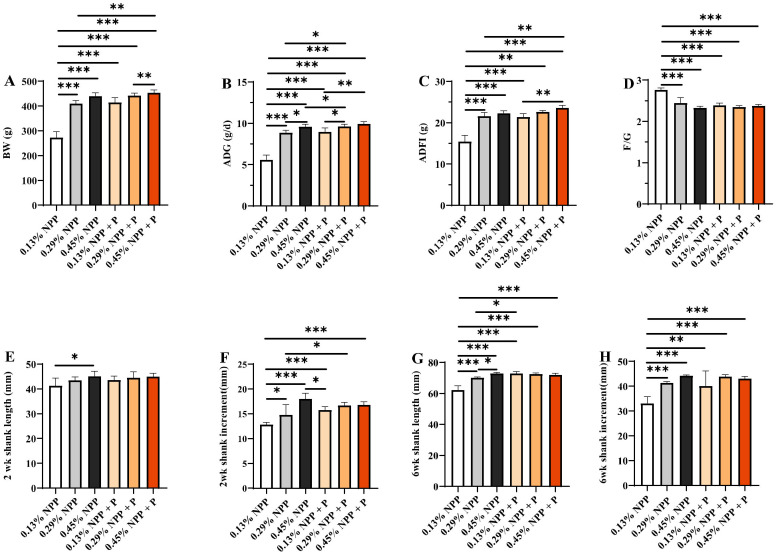
Effects of different levels of dietary phosphorus with phytase on growth performance and bone development of layers at 2 and 6 weeks of age. (**A**) BW. (**B**) ADG. (**C**) ADFI. (**D**) F/G. BW = body weight; ADG = average daily gain; ADFI = average daily feed intake; F/G = the ratio of feed to gain. (**E**) Shank length at 2 weeks of age. (**F**) Shank increment at 2 weeks of age. (**G**) Shank length at 6 weeks of age. (**H**) Shank increment at 6 weeks of age. Data are shown as means ± SEM. * *p* < 0.05, ** *p* < 0.01, *** *p* < 0.001. *n* = 6 per group.

Chicks were fed for six weeks with a series of dietary treatments consisting of three different levels of NPP (0.13%, 0.29% and 0.45%) with/without phytase. Dietary NPP and phytase levels had a significant interaction effect on average BW, ADG, ADFI and F/G (*p* < 0.01) (Figure 1 and Table S1). With the increase in phosphorus levels, BW, ADG and ADFI increased, and F/G decreased linearly (Figure S1). BW, ADG and ADFI varied significantly with different levels of phosphorus, with/without phytase (*p* < 0.001) (Figure 1). The 0.13% NPP group had significantly lower BW (*p* < 0.001), ADG (*p* < 0.001) and ADFI (*p* < 0.001) values and a significantly higher F/G (*p* < 0.001) value compared to the other treatments. Phytase supplementation in the 0.13% NPP diet improved all these growth indices, and the 0.13% NPP + P group achieved very similar growth performance to the 0.29% NPP and 0.45% NPP groups (*p* > 0.05) but still had a lower BW and ADFI than the 0.45% NPP + P group (*p* < 0.01) and a lower ADG than the other two phytase-supplemented groups (*p* < 0.05). The 0.29% NPP group exhibited a significant decrease in BW and AFDI compared with the 0.45% NPP + P group, meanwhile ADG decreased significantly compared with the 0.45% NPP, 0.29% NPP + P and 0.45% NPP + P groups (*p* < 0.05).

As shown in Figure 1 and Table S2, dietary NPP and phytase had a significant interaction effect on shank increment at 2 weeks of age and on shank length and increment at 6 weeks of age (*p* < 0.01). With the increase in phosphorus levels, shank length and shank increment increased linearly (Figure S1). At 2 weeks of age, the 0.13% NPP group showed a significant decrease in shank length and increment, and the 0.13% NPP group supplemented with phytase showed an increase in shank length and increment (*p* < 0.05). In addition, the 0.29% NPP group with phytase supplementation was also found to have an increased shank increment compared with the 0.29% NPP group (*p* < 0.05). At 6 weeks of age, the 0.13% NPP group and the 0.29% NPP group exhibited significant decreases in shank length compared with the 0.45% NPP (*p* < 0.05) and 0.13% NPP groups, and the 0.29% NPP group with phytase supplementation exhibited a significant increase in shank length (*p* < 0.05) not significantly different from that of the 0.45% NPP group (*p* > 0.05). Moreover, the shank increment in the 0.13% NPP group was significantly decreased (*p* < 0.01), while the 0.13% NPP + P group showed a significant increase in shank increment (*p* < 0.001) not significantly different from the other treatments.

### 3.2. Serum Biochemical Index

**Figure 2:**
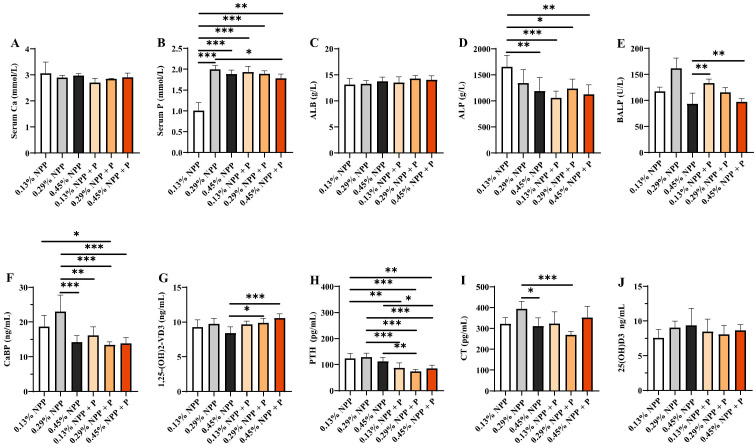
Effects of different levels of dietary phosphorus with phytase on the serum biochemical indexes of layers at 6 weeks of age. (**A**) Serum Ca. (**B**) Serum phosphorus. (**C**) ALB. (**D**) ALP. (**E**) BALP. (**F**) CaBP. (**G**) 1,25(OH)2D3. (**H**) PTH. (**I**) CT. (**J**) 25(OH)D3. Data are shown as means ± SEM. * *p* < 0.05, ** *p* < 0.01, *** *p* < 0.001. *n* = 6 per group.

As shown at Figure S2 and Table S3, dietary NPP and phytase had a significant interaction effect on serum phosphorus, ALB and ALP at 2 weeks of age (*p* < 0.01). Serum phosphorus in the 0.13% NPP group and 0.29% NPP group was significantly lower than in the 0.45% NPP group (*p* < 0.01), while, with phytase supplementation, there were significant increases in the 0.13% + P and 0.29% + P groups (*p* < 0.05). In addition, ALP content in the 0.13% NPP group was significantly increased compared with the other treatments (*p* < 0.001). The BALP content in the 0.45% NPP group was significantly higher than in the 0.13% NPP + P and 0.45% NPP + P groups (*p* < 0.01). Serum biochemical indexes of 6 weeks of age are presented in Figure 2 and Table S4; dietary NPP and phytase levels had a significant interaction effect on serum phosphorus, ALP, NBAP, CaBP, 1,25(OH)2D3 and CT (*p* < 0.01). Serum phosphorus in the 0.13% group was significantly decreased (*p* < 0.001); the addition of phytase in the 0.13% NPP diet significantly improved serum phosphorus, and there was no significant difference from other groups (*p* >0.05). In addition, the ALP content in the 0.13% NPP group was significantly higher than in the 0.45% NPP (*p* < 0.01), 0.13% NPP + P (*p* < 0.001), 0.29% NPP + P (*p* < 0.05) and 0.45% NPP + P (*p* < 0.01) groups. Moreover, we found that CaBP in the 0.29% NPP group was significantly higher than in the 0.45% NPP, 0.13% NPP + P, 0.29% NPP + P and 0.45% NPP + P groups (*p* < 0.01), and in the 0.13% NPP group was significantly higher than in the 0.29% NPP + P group (*p* < 0.05). PTH in the 0.13% NPP and 0.29% NPP groups was significantly higher than in the other groups added with phytase (*p* < 0.01), and in the 0.45% NPP group was significantly higher (*p* < 0.05) than in the 0.13% NPP + P (*p* < 0.01) and 0.45% NPP + P groups.

### 3.3. ATTD and Emission of Ca and Phosphorus

**Figure 3:**
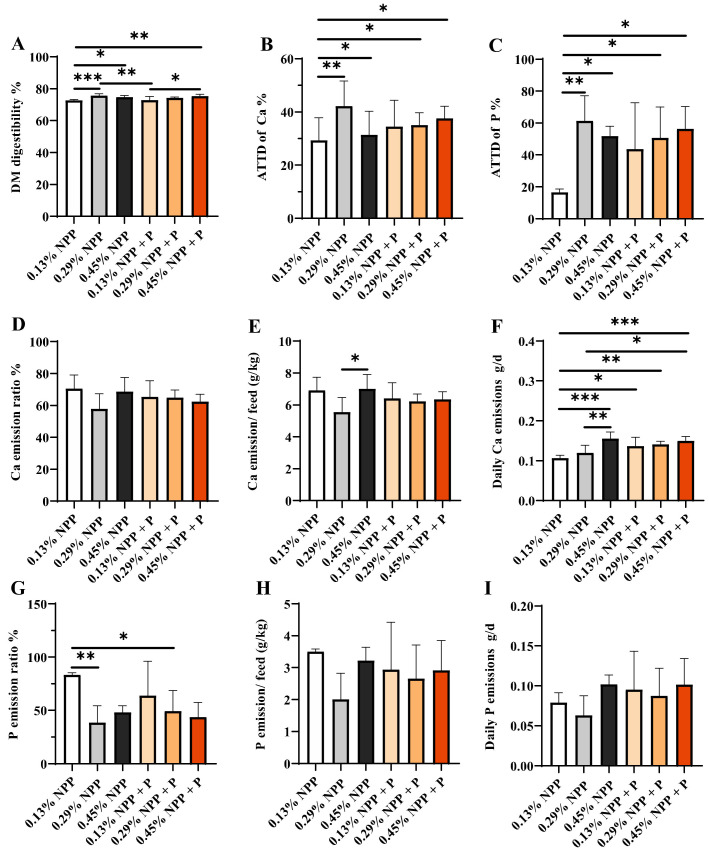
Effects of different levels of dietary phosphorus and phytase on apparent total tract digestibility (ATTD) and emission of Ca and phosphorus in layers. (**A**) DM digestibility. (**B**) ATTD of Ca. (**C**) ATTD of phosphorus. (**D**) Ca emission. (**E**) Ca emission/feed. (**F**) Daily Ca emission. (**G**) Phosphorus emission. (**H**) Phosphorus emission/feed. (**I**) Daily Ca emission. Data are shown as means ± SEM. * *p* < 0.05, ** *p* < 0.01, *** *p* < 0.001. *n* = 6 per group.

Dietary NPP and phytase levels had a significant interaction effect on Ca emission/feed and daily Ca emissions (Figure 3 and Table S5). As shown in Figure 3, the DM digestibility in the 0.13% NPP group was significantly lower than in the 0.29% NPP group and the 0.45% NPP group (*p* < 0.05); with phytase supplementation, the 0.13% NPP group still had lower DM digestibility compared to the 0.29% NPP group (Figure 3A). In addition, the ATTD of P in the 0.13% NPP group was significantly lower than in the 0.29% NPP (*p* < 0.01), 0.45% NPP (*p* < 0.05), 0.29% NPP + P (*p* < 0.05) and 0.45% NPP + P groups (*p* < 0.05), and there was no significant difference in the ATTD of phosphorus with phytase supplementation in the 0.13% NPP group (*p* > 0.05) (Figure 3C).

The Ca emission/feed in the 0.29% NPP diets was significantly lower than in the 0.45% NPP group (*p* < 0.05) (Figure 3E). The daily Ca emission in 0.13% NPP diets was significantly lower than in the 0.45% NPP (*p* < 0.001), 0.13% NPP + P (*p* < 0.05), 0.29% NPP + P (*p* < 0.05) and 0.45% NPP + P groups (*p* < 0.05) (Figure 3F). In addition, daily Ca emission in the 0.29% NPP group was significantly lower than in the 0.45% NPP group (*p* < 0.01) and in the 0.45% NPP + P group (*p* < 0.05). We also found a significant increase in the phosphorus emission ratio in the 0.13% NPP group compared with the 0.29% NPP group (*p* < 0.01) (Figure 3G). There was no significant difference in daily Ca emissions with phytase supplementation in the 0.13% and 0.29% NPP groups (Figure 3F).

### 3.4. Gut Microbial Community and Structure

16S rDNA sequencing was performed to investigate how different phosphorus and phytase levels impacted the gut microbiota composition of chicks: 3,351,225 valid sequences were obtained, 608 distinct OTUs were matched with 97% similarity and 11 phyla and 58 genera in gut microbiota were annotated. As shown in Figure S3A–D, at 2 weeks of age, no significant variations in alpha diversity were identified between treatments. However, at 6 weeks of age, the Sobs index for the 0.13% NPP group was significantly lower than it was for the 0.45% NPP (*p* < 0.05), 0.13% NPP + P (*p* < 0.001) and 0.45% NPP + P groups (*p* < 0.001), and for the 0.29% NPP group was significantly lower than for the 0.29% NPP + P group (*p* < 0.01) (Figure S3E). In addition, the ACE index for the 0.13% group was significantly lower than for the 0.45% NPP (*p* < 0.05), 0.13% NPP + P (*p* < 0.01), 0.29% NPP + P (*p* < 0.01) and 0.45% NPP + P (*p* < 0.01) groups (Figure S3G). Moreover, Chao’s index for the 0.13% group was significantly lower than it was for the 0.45% NPP (*p* < 0.01), 0.13% NPP + P (*p* < 0.05), 0.29% NPP + P (*p* < 0.01) and 0.45% NPP + P (*p* < 0.01) groups (Figure S3H).

**Figure 4:**
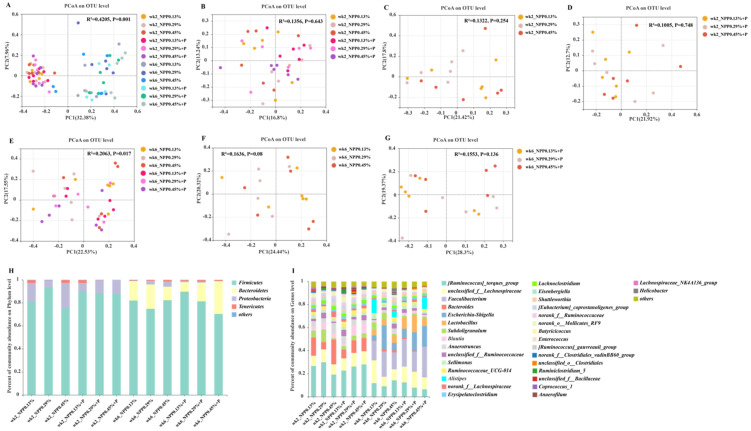
Effects of different levels of dietary phosphorus with phytase on relative abundance levels in the cecal microbiota of layers and principal coordinates analysis (PCoA, Bray–Curtis distance) plot of the gut microbial community structure. (**A**) The PCoA for the six treatments at 2 and 6 weeks of age. (**B**) The PCoA for the six treatments at 2 weeks of age. (**C**) The PCoA at different dietary phosphorus levels at 2 weeks of age. (**D**) The PCoA at different dietary phosphorus levels with phytase at 2 weeks of age. (**E**) The PCoA for the six treatments at 6 weeks of age. (**F**) The PCoA for the different dietary phosphorus levels at 6 weeks of age. (**G**) The PCoA for the different dietary phosphorus levels with phytase at 6 weeks of age. (**H**) Relative abundance of gut microbiota at the phylum level. (**I**) Relative abundance of gut microbiota at the genus level. *n* = 6 per group.

The relative abundance of organisms was studied at the phylum and genus levels. The PCoA data revealed that the microbiota structure altered between the ages of 2 and 6 weeks (Figure 4A). The distribution of spots has not presented a distinct clustering of gut microbial community structure (Figure 4B–G). Remarkably, phosphorus and phytase altered the structure and content of the chicks’ intestinal microbiota (Figure 4H,I). Interestingly, we found that *Proteobacteria* and *Firmicutes* had the highest concentrations at the phylum level at 2 weeks of age, while *Bacteroidetes* and *Firmicutes* make up the majority of bacteria in chick ceca, accounting for more than 95% of total cecal bacteria at 6 weeks of age. Cecal microbiota were dominated by *[Ruminococcus]_torques_group*, *unclassified_f__Lachnospiraceae*, *Escherichia-Shigella*, *Lactobacillus* and *Subdoligranulum* at 2 weeks of age. However, gut microbiota were dominated by *[Ruminococcus]_torques_group*, *unclassified_f__Lachnospiraceae*, *Faecalibacterium*, *Escherichia-Shigella* and *Bacteroides* at 6 weeks of age.

**Figure 5:**
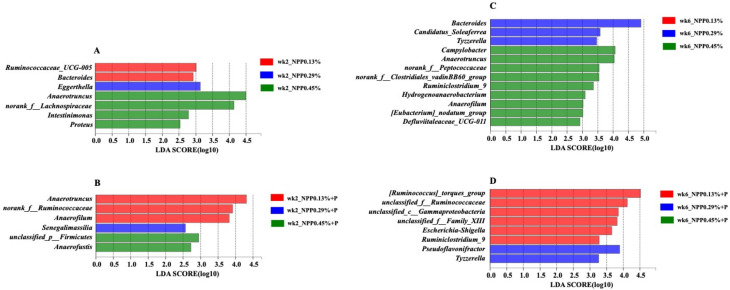
Differentially abundant genera in the gut microbiota of layers fed different levels of dietary phosphorus with phytase. (**A**) The LEfSe analysis of the gut microbiota at different dietary phosphorus levels at 2 weeks of age. (**B**) The LEfSe analysis of the gut microbiota at different dietary phosphorus levels with phytase at 2 weeks of age. (**C**) The LEfSe analysis of the gut microbiota at different dietary phosphorus levels at 6 weeks of age. (**D**) The LEfSe analysis of the gut microbiota at different dietary phosphorus levels with phytase at 6 weeks of age.

We utilized LEfSe to find out which bacterial genera were different across the groups. Relative abundance at genus level was investigated. As shown in Figure 5A, compared with the 0.13% NPP group, *Eggerthella* was enriched in the 0.29% NPP group, and *Anaerotruncus*, *norank_f__Lachnospiraceae*, *Intestinimonas* and *Proteus* were enriched in the 0.45% NPP group. As shown in Figure 5B, compared with the 0.13% NPP + P group, *Senegalimassilia* was enriched in the 0.29% NPP + P group, *unclassified_p__Firmicutes* and *Anaerofustis* were enriched in the 0.45% NPP + P group. As shown in Figure 5C, at 6 weeks of age, compared with the 0.13% NPP group, *Bacteroides*, *Candidatus_Soleaferrea* and *Tyzzerella* were enriched in the 0.29% NPP group, and *Campylobacter*, *Anaerotruncus*, *norank_f__Peptococcaceae*, *norank_f__Clostridiales_vadinBB60_group*, *Ruminiclostridium_9*, *Hydrogenoanaerobacterium*, *Anaerofilum*, *[Eubacterium]_nodatum_group* and *Defluviitaleaceae_UCG-011* were enriched in the 0.45% NPP group. As shown in Figure 5D, at 6 weeks of age, compared with the 0.13% NPP + P group, *Pseudoflavonifractor* and *Tyzzerella* were enriched in the 0.45% NPP + P group.

### 3.5. Correlation Analysis of the Gut Microbiota and Growth Performance and Phosphorus Metabolism Emission

**Figure 6:**
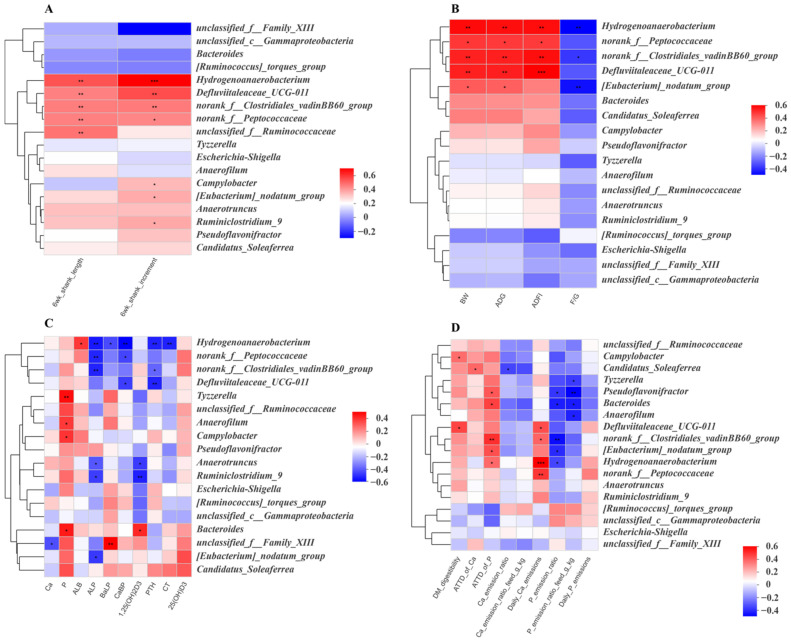
Spearman correlation analysis of the differentially abundant genera and shank growth (**A**), growth performance (**B**), serum index related to phosphorus metabolism (**C**) and Ca and phosphorus emission-related index (**D**). * *p* < 0.05, ** *p* < 0.01, *** *p* < 0.001. *n* = 6 per group.

As shown in Figure 6A, *Hydrogenoanaerobacterium*, *Defluviitaleaceae_UCG-011*, *norank_f__Clostridiales_vadinBB60_group* and *norank_f__Peptococcaceae* were positively correlated with shank length and increment. *Unclassified_f__Ruminococcaceae* was positively correlated with shank length. In addition, *Campylobacter*, *[Eubacteriucm]_nodatum_group* and *Ruminiclostridium_9* were positively correlated with shank increment. In Figure 6B, it can be seen that *Hydrogenoanaerobacterium*, *norank_f__Peptococcaceae*, *norank_f__Clostridiales_vadinBB60_group* and *Defluviitaleaceae_UCG-011* were positively correlated with BW, ADG and AFDI. *Hydrogenoanaerobacterium* and *norank_f__Clostridiales_vadinBB60_group* were negatively correlated with F/G. *[Eubacterium]_nodatum_group* was positively correlated with BW and ADG and negatively correlated with F/G.

The results of the correlational analysis of the gut microbiota and serum parameters are presented in Figure 6C. *Hydrogenoanaerobacterium* was positively correlated with ALB and negatively correlated with ALP, BALP, CaBP, PTH and CT. *Norank_f__Peptococcaceae* and *norank_f__Clostridiales_vadinBB60_group* were negatively correlated with ALP. *Norank_f__Peptococcaceae* and *Defluviitaleaceae_UCG-011* were negatively correlated with CaBP, meanwhile *norank_f__Clostridiales_vadinBB60_group* and *Defluviitaleaceae_UCG-01* were negatively correlated with PTH. *Tyzzerella, Anaerofilum*, *Campylobacter* and *Bacteroides* were positively correlated with serum phosphorus, and *Bacteroides* was positively correlated with 1,25(OH)2D3. *Anaerotruncus*, *Ruminiclostridium_9* and *[Eubacterium]_nodatum_group* were negatively correlated with ALP. Meanwhile, *Anaerotruncus* and *Ruminiclostridium_9* were negatively correlated with 1,25(OH)2D3. In addition, *unclassified_f__Family_XIII* was negatively correlated with serum Ca and positively correlated with BALP, meanwhile *[Eubacterium]_ nodatum_group* was negatively correlated with ALP.

As can be seen in Figure 6D, *Campylobacter* and *Defluviitaleaceae_UCG-011* were positively correlated with DM digestibility, and *Defluviitaleaceae_UCG-011* was positively correlated with daily Ca emission. *Candidatus_Soleaferrea* was positively correlated with ATTD of Ca and negatively correlated with Ca emission ratio. *Tyzzerella* and *Anaerofilum* were negatively correlated with P emission ratio/feed. *Pseudoflavonifractor*, *Bacteroides*, *norank_f__Clostridiales_vadinBB60_group*, *[Eubacterium]_nodatum_group* and *Hydrogenoanaerobacterium* were positively correlated with ATTD of phosphorus and negatively correlated with phosphorus emission ratio. *Pseudoflavonifractor* and *Bacteroides* were also negatively correlated with phosphorus emission ratio, while *norank_f__Clostridiales_vadinBB60_group*, *Hydrogenoanaerobacterium* and *norank_f__Peptococcaceae* were positively correlated with daily Ca emission.

## 4. Discussion

Growth performance and feed intake are essential indicators to evaluate the health status of poultry at an early age, while ADG is a sensitive indicator that reflects the utilization of phosphorus [22,23]. In the current results, the low-phosphorus diets significantly decreased growth performance in hens at 6 weeks of age; however, BW, ADG, ADFI, shank length and shank length increment were increased in the low-phosphorus diet with phytase group, which is in line with previous reports [24,25,26]. Interestingly, we found that the growth performance of the 0.45% NPP group was not influenced by phytase supplementation, which may be due to the 0.45% NPP in this group meeting the requirements for the growth and development of chicks such that additional phytase was not required to hydrolyze phytate. The meta-analysis revealed that the response of digestible phosphorus to the phytase depended on the amount of substrate in growing pigs [27]. Therefore, the current results confirm that phytase supplementation can alleviate the adverse effects of low-phosphorus diets (0.13% NPP) on growth performance, though it had a limited effect on the high-phosphorus group (0.45% NPP).

Serum phosphorus is a sensitive marker by means of which to evaluate homeostatic phosphorus in animals. In the present study, low-phosphorus diets significantly reduced serum phosphorus, and the addition of phytase significantly increased serum phosphorus and decreased ALP activity compared with the low-phosphorus diet group but had no significant effect on the adequate phosphorus treatment group, which is in accord with prior findings [28,29]. Bone phosphorus retention is involved in the skeletal development of the broiler through the regulation of ALP and BALP [30]. Chicks 1 to 42 days old are in the phase of rapid growth with high bone metabolism. ALP is a reliable biomarker for bone mineralization in birds and can reflect the activity of osteoblasts, which are at higher levels in disorders involving bone development or remodeling [31,32]. For intestinal phosphate uptake, 1,25(OH)2D3 is essential [33], increasing blood calcium and inhibiting the secretion of PTH, which is regulated by negative feedback from blood calcium. Low blood calcium levels stimulate PTH secretion, while high blood calcium prevents the release of PTH [34]. In this study, phytase supplementation significantly reduced CaBP, thereby promoting the secretion of 1,25(OH)2D3 while inhibiting the secretion of PTH. Therefore, we document that phytase prevents low-phosphorus diet-induced metabolic disorder via alterations of hormones related to phosphorus metabolism.

The gut microbiome is a complex community that influences the regulation of homeostasis in animals, playing an important role through altering host health, nutritional digestion and immunological function [35,36,37,38]. These data show differences in the composition of microbial communities, with varied influences due to different levels of phosphorus and phytase. In this study, there was no significant change in the alpha index at two weeks of age; however, low-dietary phosphorus diets decreased alpha diversity at six weeks of age. This finding is consistent with a previous study which discovered that as the host matures, the enteric microbial community becomes increasingly varied until it achieves a stable dynamic state [39]. Phosphorus is a vital nutrient for bacterial growth, bacterial structure and metabolic functions [40]. A study on pigs indicated that phosphorus might be regarded as part of an integrated approach to support immune functions and maintain a stable microbial environment, thereby providing a barrier against potential pathogenesis [2]. Furthermore, the production of fibrolytic enzymes by bacteria is highly dependent on an adequate phosphorus supply [41,42]. Therefore, these results confirm the importance of phosphorus for gut microbes; low-phosphorus diets reduce the diversity of intestinal microbial communities.

During the rearing phase, the gut microbiota was dominated by two phyla, *Firmicutes* and *Proteobacteria* [43]. Of note, the *Firmicutes*/*Bacteroidetes* ratio has been identified as a major predictor of intestinal homeostasis [44], with an increased or decreased *Firmicutes*/*Bacteroidetes* ratio indicating dysbiosis [45]. The F/B ratio decreased in the 0.45% NPP + P group in this study, which suggests that supplementation with phytase in the high-phosphorus group may also have adverse effects on gut microbiota. In addition, phosphorus deficiency caused a decrease in short chain fatty acid (SCFA) production as a result of reduced cellulose fermentation, demonstrating that the activity of bacterial fibrolytic enzymes is controlled by phosphorus [42,46]. SCFAs affect lipid and cholesterol metabolism, contributing to intestinal development in the whole growing period of broilers [47,48]. Collectively, low-phosphorus diets decreased chick growth performance in the brooding stage, which may be related to the production of SCFAs.

Numerous studies have proved that supplementation with phytase can effectively reduce fecal phosphorus emission [49,50,51]. Our results show that proper reduction of dietary phosphorus and supplementation with phytase can improve phosphorus utilization and reduce phosphorus emissions. It has been reported that high-phosphorus use efficiency cows fed low-phosphorus diets routed more endogenous phosphorus into milk and excrement., which may harm animal health and the environment in the long term [52]. Remarkably, we noticed that *Tyzzerella*, *Anaerofilum*, *Pseudoflavonifractor*, *Bacteroides, norank_f__Clostridiales_vadinBB60_group*, *Hydrogenoanaerobacterium* and *[Eubacterium]_nodatum_group* were significantly correlated with phosphorus utilization- and emission-related parameters. *Bacteroides* is beneficial in the intestinal health of poultry because of its essential role in growth performance and its fermentation products’ suppression of Clostridium perfringens sporulation [53]. In Japanese quail, based on host miRNA–mRNA and gut microbial interaction, *Bacteroides* played a vital role in phosphorus utilization [54]. Interestingly, a study has revealed that *Clostridiales vadinBB60 group* was enriched in participants who did not take vitamin D supplements after UVB exposures [55]. Combined with these results, *Bacteroides* and *norank_f__Clostridiales_vadinBB60_group* may be the key indicators of phosphorus utilization. However, since a regulation mechanism for intestinal microorganisms on phosphorus emission is not clear, further studies investigating the function of related microbiota in phosphorus utilization are required.

## 5. Conclusions

Low-phosphorus diets affected growth performance, bone development and phosphorus metabolism and altered the diversity and structure of the intestinal flora in egg-laying chicks. A combination of low-phosphorus feeding and supplementation with phytase will optimize growth performance and bone development, decrease phosphorus emission and reduce dependency on mineral phosphorus by enhancing the utilization of phytate phosphorus. These results provide new insights into phytase-induced alterations in growth performance, bone development, phosphorus metabolism and gut microbiota.

## Figures and Tables

**Table 1:** Composition and nutrient levels of experimental diets (%, air-drying basis).

Items	Treatment Groups		
	0.13% NPP	0.29% NPP	0.45% NPP
Ingredients			
Corn	65.20	65.20	65.20
Soybean meal	29.40	29.40	29.40
Dicalcium phosphate	0.00	0.95	1.90
Limestone	2.35	1.82	1.30
Zeolite powder	2.01	1.59	1.16
Salt	0.30	0.30	0.30
Choline chloride (50%)	0.10	0.10	0.10
L-Lysine HCl (98%)	0.11	0.11	0.11
DL-methionine	0.19	0.19	0.19
Premix ^1^	0.34	0.34	0.34
Total	100.00	100.00	100.00
Nutrient levels ^2^			
Metabolizable energy (MJ/kg)	11.72	11.72	11.72
Crude protein	18.00	18.00	18.00
Available methionine	0.45	0.45	0.45
Available methionine and cystine	0.74	0.74	0.74
Available Lysine	1.00	1.00	1.00
Available Tryptophan	0.20	0.20	0.20
Available Threonine	0.68	0.68	0.68
Calcium	0.98	0.96	1.02
Total phosphorus	0.42	0.51	0.67
Non-phytate phosphorus	0.13	0.29	0.45
Phytate phosphorus	0.29	0.22	0.22

^1^ Provided per kilogram of diet: vitamin A, 8000 IU; vitamin D3, 3 600 IU; vitamin E, 21 IU; vitamin K3, 4.2 mg; vitamin B1, 3 mg; vitamin B2, 10.2 mg; folic acid, 0.9 mg; pantothenic acid, 15 mg; niacin, 45 mg; vitamin B6, 5.4 mg; vitamin B12, 24 μg; biotin, 0.15 mg; copper, 6.8 mg; iron, 66 mg; zinc, 83 mg; manganese, 80 mg; iodine, 1 mg; Se 0.3 mg. ^2^ All nutrient levels were calculated, except calcium and total phosphorus were measured values.

## Data Availability

The data presented in this study are available on request from the corresponding author.

## Supplementary Materials

The following are available online at https://www.mdpi.com/article/10.3390/ani12070940/s1, Figure S1: Regression equation between different levels of dietary phosphorus and growth performance and bone development with or without phytase in layers, Figure S2: Effects of different levels of dietary phosphorus with phytase on serum biochemical indexes of layers at 2 weeks of age, Figure S3: Effects of different levels of dietary phosphorus with phytase on the alpha diversity of layers, Table S1: Effects of interaction between different levels of phosphorus and phytase on the growth performance of chicks during the brooding period, Table S2: Effects of interaction between different levels of phosphorus and phytase on shank length and shank increment in chicks during the brooding period, Table S3: Effects of interaction between different levels of phosphorus and phytase on serum indexes of chicks at 2 weeks of age, Table S4: Effects of interaction between different levels of phosphorus and phytase on serum indexes of chicks at the end of 6 weeks of age, Table S5: Effects of interaction between different levels of phosphorus and phytase on apparent total digestibility and emission of Ca and P in chicks during the brooding period.
