# Supplementary material for: Growth Performance, Bone Development and Phosphorus Metabolism in Chicks Fed Diets Supplemented with Phytase Are Associated with Alterations in Gut Microbiota
Source: Animals (Basel). 2022 Apr 6;12(7):940. doi: 10.3390/ani12070940 (PMC8997062; doi:10.3390/ani12070940)
Supplement: Supplementary file 1 [file animals-12-00940-s001.zip › Supplementary materials.pdf]

Supplemental Material

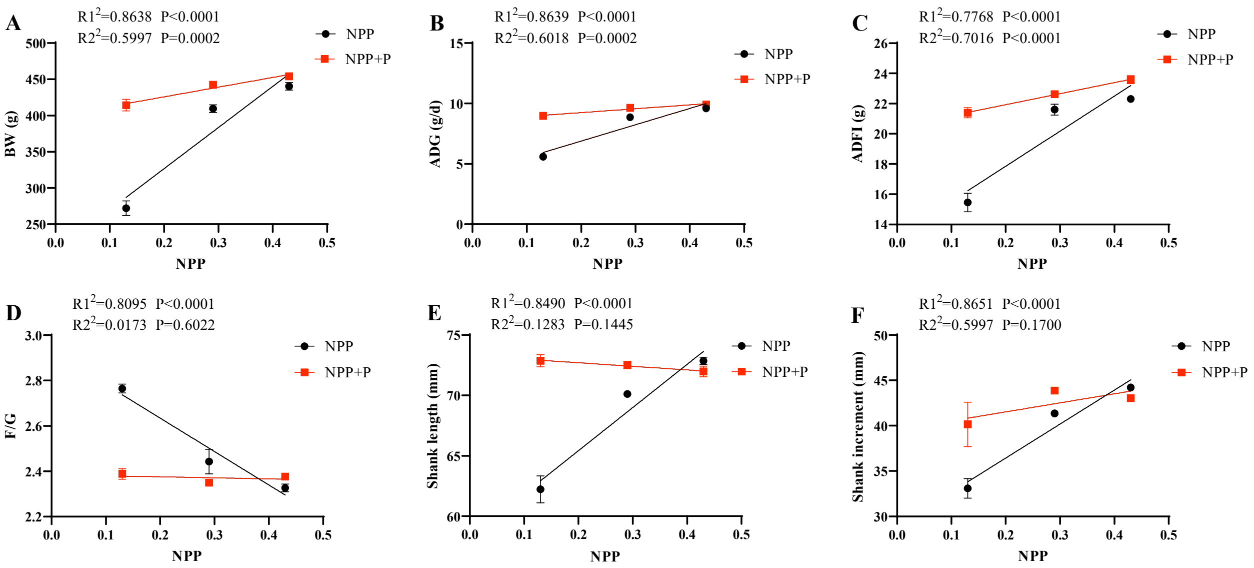

Figure S1. Regression equation between different levels of dietary phosphorus and growth performance and bone development with or without phytase of layers. (A) BW (B)ADG (C) ADFI (D) F/G (E) Shank length (F) Shank increment. BW = body weight, ADG = average daily gain; ADFI = average daily feed intake; F/G = the ratio of feed to gain. Data are shown as mean ± SEM. n = 6 per group.

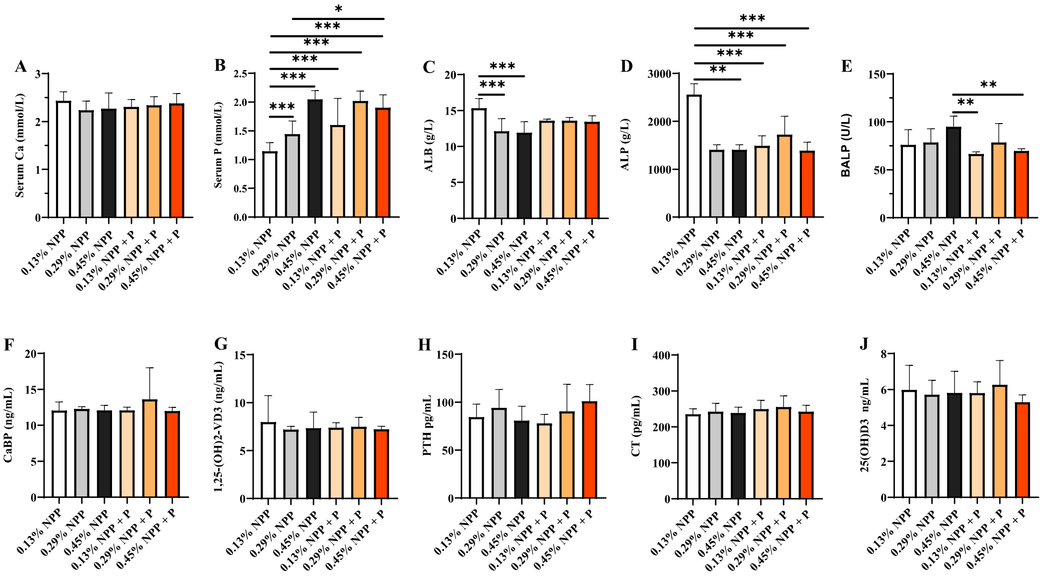

Figure S2. Effects of different levels of dietary phosphorus with phytase on serum biochemical index of layers at 2 weeks of age. (A) Serum Ca (B) Serum phosphorus (C) ALB (D) ALP (E) BALP (F) CaBP (G) 1,25(OH)2D3 (H) PTH (I) CT (J) 25(OH)D3. Data are shown as mean ± SEM. * $P < 0.05$, ** $P < 0.01$, *** $P < 0.001$, ****$P < 0.0001$. $n = 6$ per group.

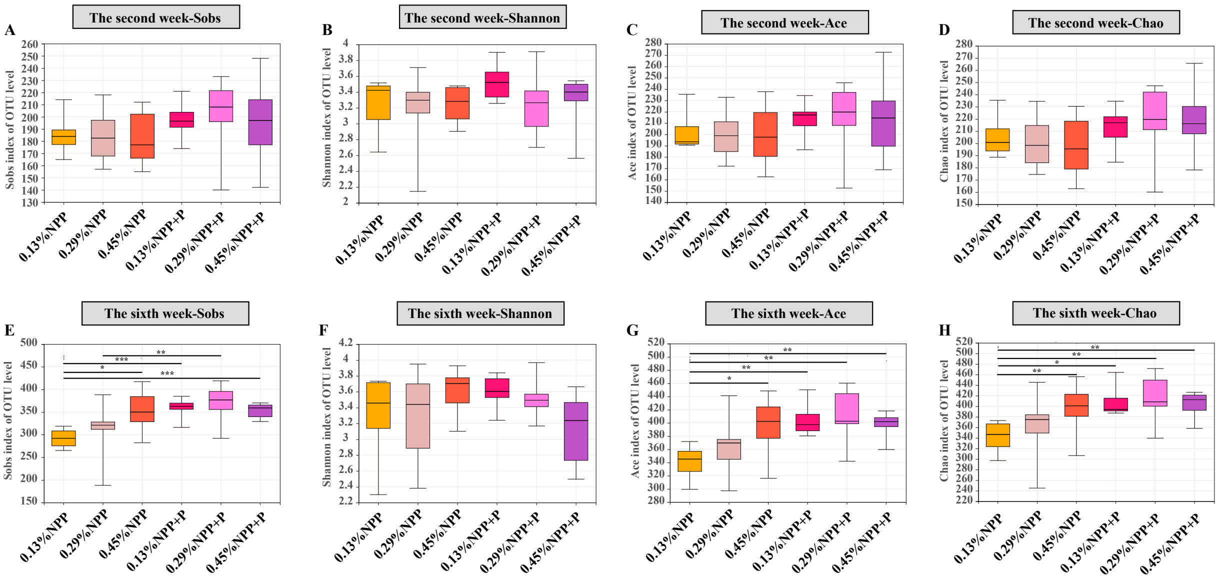

Figure S3. Effects of different levels of dietary phosphorus with phytase on alpha diversity of layers. (A) Sob index at 2 weeks of age (B) Shannon index at 2 weeks of age (C) Ace index at 2 weeks of age (D) Chaos index at 2 weeks of age (E) Sob index at 6 weeks of age (F) Shannon index at 6 weeks of age (G) Ace index at 6 weeks of age (H) Chaos index at 2 weeks of age Data are shown as mean ± SEM. * $P < 0.05$, ** $P < 0.01$, *** $P < 0.001$. n = 6 per group.

Table S1. Effects of interaction between different levels of phosphorus and phytase on growth performance of chicks during brooding period

Items		BW g	ADG g/d	ADFI g/d	F/G
NPP	0.13%	343.23[c]	7.28[c]	18.42[c]	2.58[a]
	0.29%	425.96[b]	9.25[b]	22.17[b]	2.40[b]
	0.45%	447.15[a]	9.75[a]	22.94[a]	2.35[b]
Phytase	0	374.00[b]	8.01[b]	19.79[b]	2.51[a]
	200	436.90[a]	9.51[a]	22.54[a]	2.37[b]
SEM		6.25	0.16	0.36	0.03
P-value	NPP	<0.01	<0.01	<0.01	<0.01
	Phytase	<0.01	<0.01	<0.01	<0.01
	NPP×Phytase	<0.01	<0.01	<0.01	<0.01

Data are shown as mean ± SEM. $n = 6$ per group. Different letters signify significantly different groups ($P < 0.05$). BW = body weight, ADG = average daily gain; ADFI = average daily feed intake; F/G = the ratio of feed to gain.

Table S2. Effects of interaction between different levels of phosphorus and phytase on shank length and shank increment of chicks during brooding period

Items		2wk		6wk	
		Shank length mm	Shank increment mm	Shank length mm	Shank increment mm
NPP	0.13%	42.33^{b}	14.31^{c}	67.55^{b}	36.62^{b}
	0.29%	43.96^{ab}	15.75^{b}	71.32^{a}	42.62^{a}
	0.45%	45.00^{a}	17.41^{a}	72.41^{a}	43.63a
Phytase	0	43.26	15.22	68.40^{b}	39.55
	200	44.37	17.40	72.46^{a}	42.35
SEM		0.39	0.33	0.57	0.55
P-value	NPP	0.02	<0.01	<0.01	<0.01
	Phytase	0.14	<0.01	<0.01	<0.01
	NPP×Phytase	0.15	<0.01	<0.01	<0.01

Data are shown as mean ± SEM. $n = 6$ per group. Different letters signify significantly different groups ($P < 0.05$).

Table S3. Effects of interaction between different levels of phosphorus and phytase on serum indexes of chicks at 2 weeks of age

Items		Ca mmol/L	P mmol/L	ALB g/L	ALP U/L	BALP U/L	CaBP ng/ml	$1,25(OH)_2D_3$ ng/ml	PTH pg/ml	CT pg/ml	$25(OH)D_3$ ng/ml
NPP	0.13%	2.37	1.37^{b}	14.44^{a}	2025.40^{a}	71.19	12.07	7.69	81.19	242.22	5.89
	0.29%	2.29	1.73^{a}	12.84^{b}	1562.77^{b}	78.58	12.96	7.34	92.27	248.79	5.99
	0.45%	2.32	1.97^{a}	12.67^{b}	1398.15^{b}	82.27	12.04	7.28	90.88	240.54	5.56
Phytase	0	2.31	1.54^{b}	13.11	1789.46^{a}	83.18^{a}	12.14	7.51	86.43	238.78	5.83
	200	2.34	1.84^{a}	13.53	1534.75^{b}	71.52^{b}	12.56	7.37	89.80	248.92	5.79
SEM		0.08	0.10	0.42	81.20	4.44	0.51	0.45	7.00	8.73	0.39
P-value	NPP	0.66	<0.01	<0.01	<0.01	0.11	0.41	0.75	0.28	0.63	0.57
	Phytase	0.69	<0.01	0.29	<0.01	0.01	0.51	0.77	0.58	0.18	0.89
	NPP× Phytase	0.31	<0.01	<0.01	<0.01	0.06	0.59	0.74	0.16	0.81	0.44

Data are shown as mean ± SEM. $n = 6$ per group. Different letters signify significantly different groups ($P < 0.05$). ALB=Albumin, ALP=Alkaline Phosphatase, BALP= Bone Alkaline Phosphatase, CaBP=Calcium Binding Protein, CT=Calcitonin, PTH= Parathyroid Hormone.

Table S4. Effects of interaction between different levels of phosphorus and phytase on serum indexes of chicks at the end of 6 weeks of age

Items		Ca mmol/L	P mmol/L	ALB g/L	ALP U/L	BALP U/L	CaBP ng/ml	1,25(OH)$_2$D$_3$ ng/ml	PTH pg/ml	CT pg/ml	25(OH)D$_3$ ng/ml
NPP	0.13%	2.89	1.47^{b}	13.31	1356.71	125.55^{b}	17.44^{a}	9.47	105.84	322.82	8.01
	0.29%	2.88	1.95^{a}	13.76	1289.37	138.85^{a}	18.21^{a}	9.80	101.42	331.70	8.57
	0.45%	2.94	1.84^{a}	13.89	1156.79	95.14^{c}	14.02^{b}	9.50	99.30	331.69	9.00
Phytase	0	2.98^{a}	1.16^{b}	13.37	1394.33^{a}	124.20	18.63^{a}	3.14^{b}	121.79^{a}	342.35	8.65
	200	2.82^{b}	1.87^{a}	13.40	1140.91^{b}	115.50	14.49^{b}	10.04^{a}	82.59^{b}	315.12	8.40
SEM		0.06	0.05	0.35	84.22	4.83	1.01	0.03	5.9	15.85	0.58
P-value	NPP	0.69	<0.01	0.25	0.08	<0.01	<0.01	0.51	0.56	0.83	0.30
	Phytase	0.03	<0.01	0.06	<0.01	0.06	<0.01	<0.01	<0.01	0.06	0.63
	NPP× Phytase	0.13	<0.01	0.56	<0.01	<0.01	<0.01	<0.01	0.08	<0.01	0.29

Data are shown as mean ± SEM. n = 6 per group. Different letters signify significantly different groups ($P < 0.05$). ALB=Albumin, ALP=Alkaline Phosphatase; BALP= Bone Alkaline Phosphatase, CaBP=Calcium Binding Protein, CT=Calcitonin, PTH= Parathyroid Hormone.

Table S5. Effects of interaction between different levels of phosphorus and phytase on Apparent Total Digestibility and emission of Ca and P of chicks during brooding period

Items		DM digestibility %	ATTD of Ca %	ATTD of P %	Ca emission ratio %	Ca emission/feed g/kg	Daily Ca emissions g/d	P emission ratio %	P emission/feed g/kg	Daily P emissions g/d
NPP	0.13%	72.82[b]	32.16	30.90[b]	67.83	6.65[a]	0.12	71.70[a]	3.16[a]	0.09[ab]
	0.29%	75.08[a]	38.58	58.36[a]	61.42	5.90[b]	0.13	43.93[b]	2.33[b]	0.08[b]
	0.45%	75.09[a]	34.49	54.17[a]	65.51	6.68[a]	0.15	45.82[b]	3.07[ab]	0.10[a]
Phytase	0	74.43	34.29	3.32	65.42	6.47	0.13	53.35	2.84	0.08
	200	74.23	35.71	52.30	64.29	6.34	0.14	52.26	2.84	0.09
SEM		0.42	3.06	5.45	1.42	0.15	0.003	5.90	0.29	0.01
P value	NPP	<0.01	0.15	<0.01	0.15	0.03	<0.01	<0.01	0.07	0.13
	Phytase	0.61	0.60	0.49	0.60	0.57	<0.01	0.49	0.83	0.22
	NPP×Phytase	0.08	0.09	0.18	0.09	0.01	0.02	0.18	0.28	0.61

Data are shown as mean ± SEM. n = 6 per group. Different letters signify significantly different groups ($P < 0.05$). DM= dry matter; ATTD = Apparent Total Tract Digestibility.
